# Meta-analysis of the rs243865 MMP-2 polymorphism and age-related macular degeneration risk

**DOI:** 10.1371/journal.pone.0213624

**Published:** 2019-03-07

**Authors:** Ricardo Usategui-Martín, Salvador Pastor-Idoate, Antonio J. Chamorro, Itziar Fernández, Iván Fernández-Bueno, Miguel Marcos-Martín, Rogelio González-Sarmiento, José Carlos Pastor

**Affiliations:** 1 Instituto Universitario de Oftalmobiología Aplicada (IOBA), University of Valladolid, Valladolid, Spain; 2 Departament of Ophthalmology, Hospital Clínico Universitario de Valladolid, Valladolid, Spain; 3 Red Temática de Investigación Cooperativa en Salud (RETICS), Oftared, Instituto de Salud Carlos III, Valladolid, Spain; 4 Department of Internal Medicine, University Hospital of Salamanca-SACYL, Salamanca, Spain; 5 Departament of Medicine, University of Salamanca, Salamanca, Spain; 6 Department of Statistics and Operative Research, University of Valladolid, Valladolid, Spain; 7 Institute of Biomedical Research of Salamanca (IBSAL), Salamanca, Spain; 8 Bioengineering, Biomaterials and Nanomedicine (CIBER-BBN), Valladolid, Spain; 9 Centro en Red de Medicina Regenerativa y Terapia Celular de Castilla y León, Valladolid, Spain; 10 Institute of Molecular and Cellular Biology of Cancer (IBMCC), University of Salamanca-CSIC, Salamanca, Spain; Massachusetts Eye & Ear Infirmary, Harvard Medical School, UNITED STATES

## Abstract

**Purpose:**

Several researchers have suggested that the rs243865 (16q13-q21) polymorphism in the promoter region of the metalloproteinase-2 (MMP-2) gene could be associated with an increased risk of developing age-related macular degeneration (AMD). However, previous results remain inconclusive. To clarify this controversy, we conducted a meta-analysis of the relationship between rs243865 of MMP-2 and AMD.

**Methods:**

We included 6 independent case-control studies involving 1,682 AMD patients and 2,295 healthy subjects. The association between the rs243865 polymorphism and AMD was examined by the overall odds ratio (OR) with a 95% confidence interval (CI). We used a recessive genetic model analysis, sensitivity analysis, and assessment of bias in our meta-analysis.

**Results:**

Our results showed that there was no significant association between the variant T allele (p-value = 0.10, OR [95%CI] = 0.95 [0.82–1.10]) or the CT+TT genotype (p-value = 0.16, OR [95%CI] = 0.92 [0.76–1.12]) of rs243865 MMP-2 polymorphism and the presence of AMD.

**Conclusions:**

The rs243865 MMP-2 polymorphism was not associated with an increased risk of developing AMD. The MMP-2 (-1306 C>T) promoter variant is unlikely to have a major role in AMD risk susceptibility.

## Introduction

Age-related macular degeneration (AMD) is the leading cause of severe vision loss and blindness worldwide, and the incidence in developed countries ranges from 9% to 25% at ages 65 to 75 years [1,2]. The risk of developing advanced AMD lesions increases with age and is ten-fold greater in people over 85 years compared those 65 to 75 years old [3]. According to the severity of the disease, AMD is classified as early, intermediate, and advanced. The advanced form includes atrophic and/or neovascular conditions [4].

The development of AMD is influenced by lifestyle (smoking), dietary factors, drusen accumulation, and parainflammation. In addition, the incidence of patients with AMD is also affected by genetic components [5,6]. Current data suggests that the rs243865 matrix metalloproteinase-2 (MMP-2) polymorphism within the 16q13-q21 chromosomal region is associated with an increased risk of developing AMD [7–12]. The rs243865 polymorphism is located in the promoter transcription region of the MMP-2 gene, and it consists of a C>T change (-1306C>T). This modifies the promoter activity of the MMP-2 gene, altering the Sp1-type promoter site [13]. The MMP-2 gene is crucial for the regulation of extracellular matrix components [14,15], and extracellular matrix dysregulation could be associated with the development of AMD [5]. In the studies of allelic association with AMD, the rs243865 MMP-2 polymorphism has been especially analyzed because it modifies MMP-2 activity. Several groups have reported the association between the rs243865 MMP-2 polymorphism and the risk of AMD [7–12]. However, the results from previous studies have been inconclusive, partly because each study was underpowered due to inadequate sample sizes. We have chosen to analyze the rs243865 polymorphism for two main reasons: (1) it modifies the MMP-2 gene promoter activity and, thus, the extracellular matrix regulation, which may be linked to the development of AMD [5]; and (2) because to the best of our knowledge, the rs243865 MMP-2 polymorphism has not been included in any genome-wide association (GWAS) or meta-analysis studies for AMD. Thus, we conducted this meta-analysis based on the published literature to characterize more precisely the potential association between the rs243865 polymorphism and AMD.

## Materials and methods

The current study conformed to the checklist for meta-analysis of genetic association studies specified for PLOS One approach (S1 Table) [16].

### Search strategy and selection criteria

To identify eligible studies, we searched in the PubMed, Web of Science, Scopus, and Embase electronic databases. Potentially relevant articles were sought by using the search terms in combination as Medical Subject Headings (MeSH) terms and text words: “matrix metalloproteinase-2”, “polymorphism”, “rs243865”, “-1306C>T”, “age-related macular degeneration”, “AMD”, “macular degeneration”, and “maculopathy”. We also scanned the reference lists of the retrieved publications to identify additional relevant articles (S2 Table).

The following inclusion criteria were used to identify published articles for our meta-analysis: (1) associations between the rs243865 MMP-2 polymorphism and AMD published in the last 18 years; (2) study populations included only individuals not related to one another; (3) sufficient genotype data were presented to allow calculation of the odds ratios (ORs); (4) each study clearly described the diagnosis of AMD, which was limited to the National Institute for Health and Care Excellence (NICE) Age-Related Maculopathy grading system or the International Classification and Grading system [17,18]; (5) the sources of the cases and controls were identified; and (6) genotype distributions complied with the Hardy–Weinberg equilibrium (HWE). In addition, we excluded reviews and redundant studies. Only English-language articles were analysed.

### Data extraction

To improve the reliability of our results, two investigators (ALP, MML) used a standardised form to independently extract data. Any divergences of opinion were resolved by consensus with the senior authors (RGS, JCP). Second and third literature searches were conducted but did not contribute additional studies for the analyses. Citations were first scanned at the title/abstract level. Shortlisted studies were then retrieved in full text.

From each study, the following information was extracted: the author´s name, year of publication, location, and ethnicity and demographic information (age and sex) of the study population. Allele and genotype frequencies were extracted or calculated. HWE was verified for each eligible study. The corresponding authors of the original studies were contacted if further data were needed.

### Quality assessment

Study quality was ranked as high, moderate, or low (score categories 7–9, 4–6, 0–3, respectively) by the Newcastle-Ottawa Quality Assessment Scale (NOS) [19]. Two independent reviewers conducted the data extraction and quality assessment, and disagreements were resolved by discussion (S3 Table).

### Statistical analyses

The main meta-analysis compared the presence of the variant T allele of rs243865 MMP-2 polymorphism among AMD patients as cases versus healthy subjects as controls. If three or more studies were available, further meta-analyses were performed comparing: (1) the presence of CT+TT genotype of rs243865 MMP-2 polymorphism among AMD patients and healthy subjects; (2) the presence of CT+TT genotype of rs243865 MMP-2 polymorphism among AMD patients and healthy subjects by age; and (3) the presence of CT+TT genotype of rs243865 MMP-2 polymorphism among AMD patients > 65 years old and AMD patients < 65 years old.

For each study, we used a random-effects model [20] to calculate the OR and the 95% confidence interval (CI) along with the respective p-values. P-values < 0.05 were considered statistically significant. To evaluate heterogeneity, Cochran´s Q-statistic was used. For estimation of inconsistency in meta-analysis, the I^2^ statistic was determined, representing the percentage of the observed study variability attributed to heterogeneity rather than to chance [21]. Begger´s funnel plots were drawn to assess publication bias. Sensitivity analysis was performed by excluding individual studies. The meta-analysis was performed using RevMan 5.0 software [22]. Deviation of genotype frequencies from the HWE in healthy subjects was assessed by X^2^ test.

## Results

### Characteristics of the included studies

**Fig 1:**
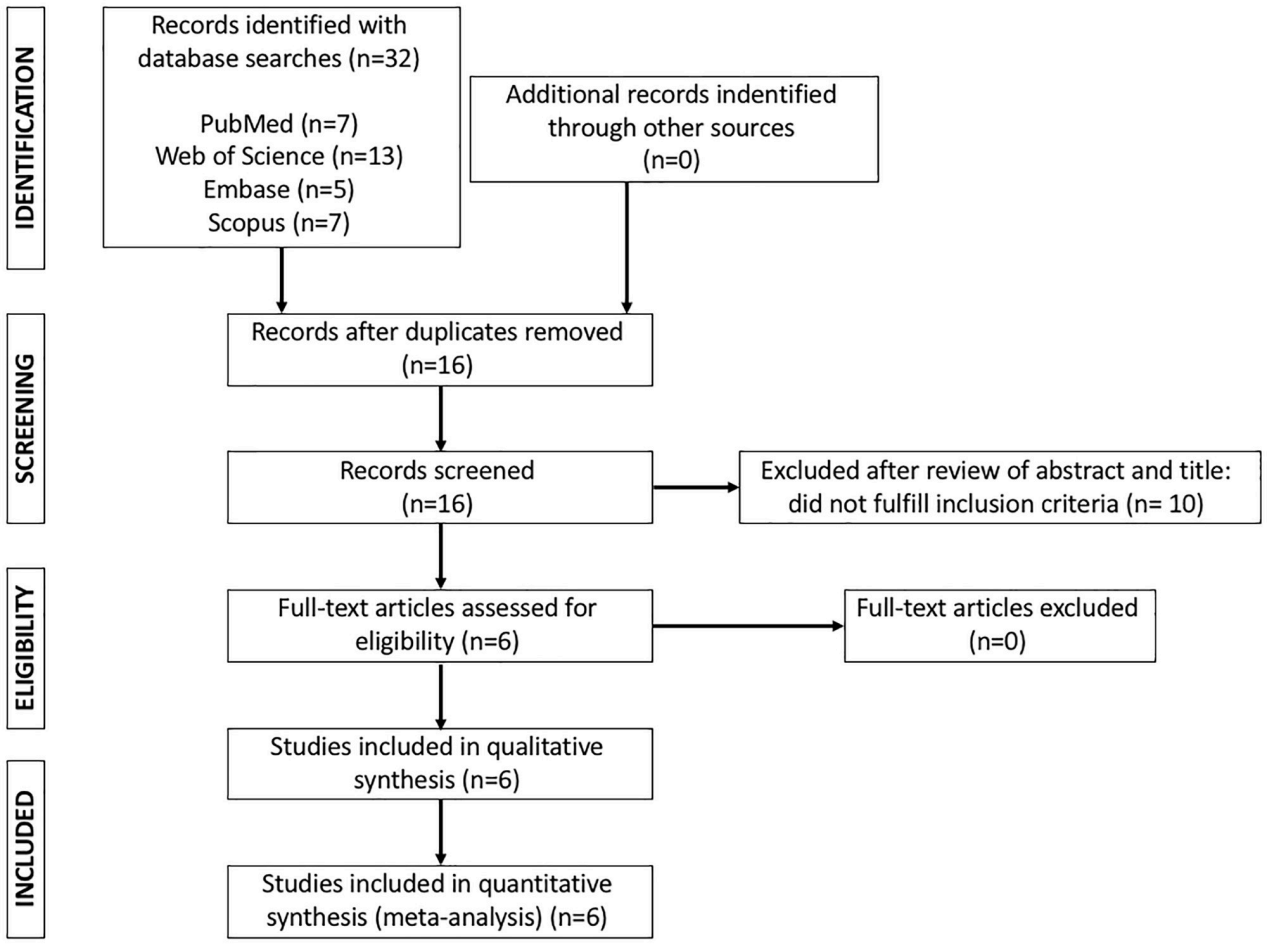
Flow chart of the selection of studies for inclusion in the meta-analysis.

A flow diagram of the study selection process is shown in Fig 1. The initial search identified 32 references for possible inclusion in our meta-analysis. After removing duplicates, 16 studies remained, and 10 of these were excluded after abstract examination revealed that they failed to meet the inclusion criteria (S2 Table). This step left 6 full articles that were assessed for eligibility [7–12], and none of them had to be removed. The corresponding author of three articles [9,11,12] was contacted due to doubts about a possible overlap of the patients and controls included in the articles. The corresponding author in each case confirmed to us that the patients and controls included in each article were different, so that the three articles were included in the meta-analysis. Therefore, a total of 6 studies were ultimately included (Fig 1). These studies included 1,682 AMD patients and 2,295 healthy subjects, and the population demographics are shown Table 1.

**Table 1:** Characteristics of the studies included in the meta-analysis.

Authors, year	Country	Ethnicity	Female/male ratio		Age, y [mean ± SD or median (min—max)]		HWE p-value
			AMD patients	Healthy subjects	AMD patients	Healthy subjects	
Cheng et al., 2017	China	Asian	42/84	50/91	62.4±9.6	63.8±10.2	0.117
Liutkeviciene et al., 2016	Lithuania	Caucasian	186/201	330/352	65 (57–93)	66 (55–97)	0.664
Ortak et al., 2013	Turkey	N/A	79/65	84/88	67.1 ± 6.9	66.1 ± 6.5	>0.05
Seitzman et al., 2008	USA	Caucasian	434/0	456/0	> 60	>60	>0.05
Liutkeviciene et al., 2017	Lithuania	Caucasian	164/126	316/210	67 (59.9–67.8)	68 (60.8–68.3)	0.528
Liutkeviciene et al., 2018	Lithuania	Caucasian	168/99	205/113	75.4±7.6	74.7±8.4	0.382

AMD: Age-related macular degeneration; N/A: not available; y: years; SD: standard deviation; HEW: Hardy-Weinberg equilibrium.

### Meta-analysis databases: Association between rs243865 MMP-2 polymorphism and AMD

Each of the articles analysed in our meta-analysis included the genotypic and allelic frequencies of rs243865 MMP-2 polymorphism in AMD patients and healthy controls (Table 2). All of the studies used genomic DNA extracted from nucleated peripheral blood cells, and genotyping was performed with polymerase chain reaction-restriction fragment length polymorphism (PCR-RFLP). There were no statistically significant deviations from the HWE in the control groups of any of the studies.

**Table 2:** Genotype and allelic distribution of rs243865 MMP-2 polymorphism in age-related macular degeneration patients and healthy subjects.

Authors, year	Subject groups	N	Genotype distribution				Allele distribution	
			CC	CT	TT	CT+TT	C	T
Cheng et al., 2017	AMD patients	126	94	27	5	32	215	37
	Healthy subjects	141	89	42	10	52	220	62
	AMD patients < 65 y	53	40	N/A	N/A	13	N/A	N/A
	Healthy controls < 65 y	76	49	N/A	N/A	27	N/A	N/A
	AMD patients > 65 y	73	54	N/A	N/A	19	N/A	N/A
	Healthy controls > 65 y	65	40	N/A	N/A	25	N/A	N/A
Liutkeviciene et al., 2016	AMD patients	387	229	132	26	158	590	184
	Healthy controls	682	385	252	45	297	1022	342
	AMD patients < 65 y	181	118	48	15	63	284	78
	Healthy controls < 65 y	306	186	103	17	120	475	137
	AMD patients > 65 y	206	111	84	11	95	306	106
	Healthy controls > 65 y	376	199	149	28	177	547	205
Ortak et al., 2013	AMD patients	144	80	60	4	64	220	68
	Healthy controls	172	108	64	0	64	280	64
Seitzman et al., 2008	ADM patients	434	N/A	N/A	N/A	N/A	660	208
	Healthy controls	456	N/A	N/A	N/A	N/A	678	234
Liutkeviciene et al., 2017	ADM patients	324	191	112	21	133	494	154
	Healthy controls	526	289	198	39	237	776	276
Liutkeviciene et al., 2018	AMD patients	267	157	84	26	110	398	136
	Healthy controls	318	190	108	20	128	488	148
	AMD patients < 65 y	99	56	35	8	43	147	51
	Healthy controls < 65 y	113	69	39	5	44	177	49
	AMD patients > 65 y	168	101	49	18	67	251	85
	Healthy controls > 65 y	205	121	69	15	84	311	99

MMP-2, matrix metalloproteinase-2 gene; AMD, age-related macular degeneration; y, years of age; N/A, not available

**Fig 2:**
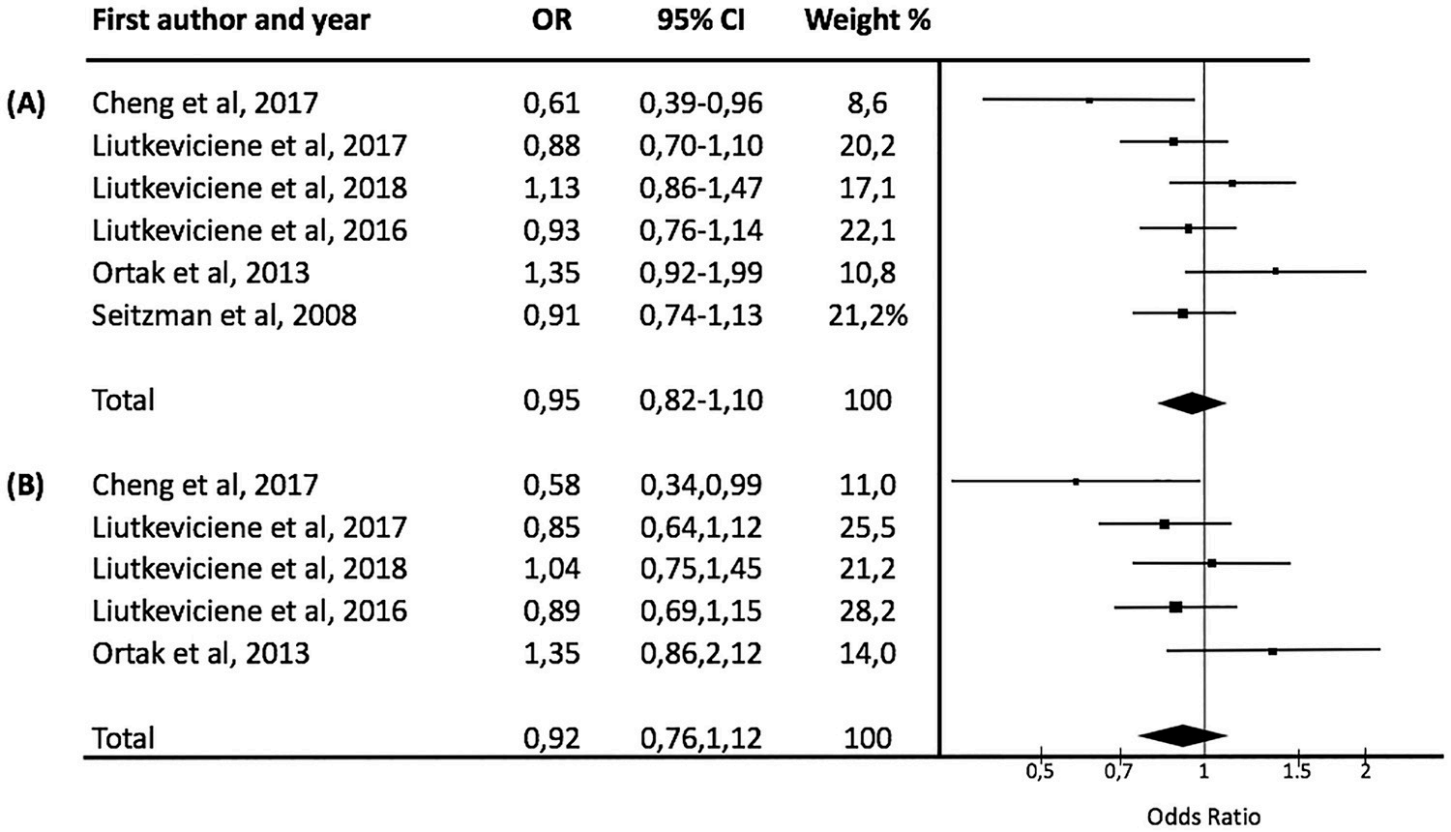
Meta-analysis of the association between rs243865 MMP-2 polymorphism and age-related macular degeneration. (A) C vs. T allele. Test for overall effect: Z = 0.67 (P = 0.50). Test for heterogeneity: v2 = 9.17 (P = 0.10). Test for inconsistency: I^2^ = 46%. (B) CC vs. CT+TT genotype. Test for overall effect: Z = 0.82 (P = 0.41). Test for heterogeneity: v2 = 6.55 (P = 0.16), Test for inconsistency: I^2^ = 39%. Each study is shown by an OR estimate with the corresponding 95% CI.

After analysis of the pooled selected studies, there were no significant associations between the variant T allele (Fig 2A, p-value = 0.10, OR [95%CI] = 0.95 [0.82–1.10]) or the CT+TT genotype (Fig 2B, p-value = 0.16, OR [95%CI] = 0.92 [0.76–1.12]) of the rs243865 MMP-2 polymorphism and the presence of AMD (Fig 2). There were also no significant differences based on study population ethnicity. The sensitivity analysis, which excluded individual studies, did not modify these results. Further, there were no significant differences in the genotypic distribution of rs243865 MMP-2 polymorphism based on age between AMD patients and healthy subjects (Table 3). Nor was there a difference in the genotypic distribution between AMD patients > 65 years old and those < 65 years old (Table 3).

**Table 3:** Summary of meta-analysis results for the association of rs243865 MMP-2 polymorphism with age-related macular degeneration by age.

	OR	95% CI	P (random-effects model)	I^2^	P (heterogeneity)
**AMD patients < 65 y vs healthy subjects < 65 y (N = 3)**					
CC vs CT+TT	0.88	0.66–1.17	0.44	15%	0.31
**AMD patients > 65 y vs healthy subjects > 65 y (N = 3)**					
CC vs CT+TT	0.90	0.70–1.15	0.41	0%	0.40
**AMD patients > 65 y vs AMD patients < 65 y (N = 3)**					
CC vs CT+TT	1.19	0.78–1.81	0.43	44%	0.43

OR, odds ratio; CI, confidence interval; AMD, age-related macular degeneration; y, age in years; N, number of studies included in each meta-analysis.

### Bias diagnostics

**Fig 3:**
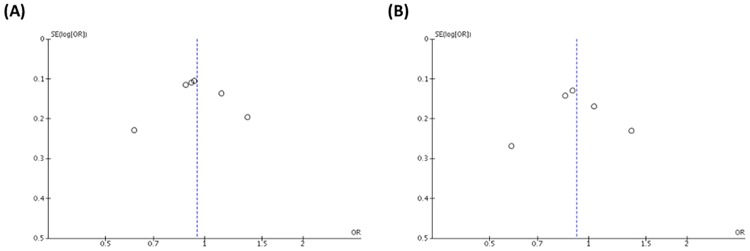
Funnel plot of studies included in the meta-analysis assessing the association of the rs243865 MMP-2 polymorphism with age-related macular degeneration. The effect size (OR) was plotted on the x-axis, and the inverse of variance of the effect was plotted on the y-axis. (A) C vs. T allele. (B) CC vs. CT+TT genotype.

When there are fewer than 10 studies in a meta-analysis, the power of tests for funnel plot asymmetry is too low to distinguish chance from real asymmetry [23]. Even so, we examined publication bias by visual inspection using a Begger’s funnel plot with the effect size, i.e., the OR, on the *x*-axis and the inverse of variance of the effect on the *y*-axis (Fig 3). The shape of the funnel plot for publication bias appeared to be symmetrical (Fig 3A and 3B), although there was some uncertainty regarding the degree of symmetry. Thus, the estimate of the effect was considered to be biased. Nevertheless, the results based on the Begger’s test of the allele (C vs. T, Fig 3A) and the recessive (CC vs. CT/TT, Fig 3B) models showed no evidence of publication bias.

### Sensitivity analyses

Sensitivity analyses were performed after sequentially removing each eligible study. This approach is regarded as an indispensable step for analysing multiple criteria. The significance of the pooled ORs was not influenced by any single study in the recessive genetic model, indicating that our results were statistically robust.

## Discussion

The aetiology of AMD is unknown; nevertheless, several observations suggest that genetic alterations may be crucial in the development of the disease [5,6]. To date, through GWAS, more than 50 independently associated common genetic variants distributed across more than 30 loci have been associated with a significantly increased susceptibility to developing AMD [24–26]. To our knowledge, none of them included an analysis of the rs243865 MMP-2 polymorphism. In particular, variations in the complement factor H gene and the ARMS2/HTRA1 genes have been considered to be the most consistent genetic risk factors for AMD development [27,28]. The precise genetic factors, as well as their interactions, remain unclear in AMD. Therefore, in an attempt to prevent AMD, the identification of other potential genetic factors is crucial due to the lack of effective treatments at present.

AMD is characterised by drusen formation, which are accumulations of abnormal extracellular matrix deposits that cause visual function impairment [29,30]. A growing number of studies suggest that the rs243865 MMP-2 polymorphism could be associated with increased susceptibility to AMD development [7–12]. However, the results from past studies remain inconclusive, with some studies supporting a significant association, while others claim the opposite. Also, caution should be undertaken when interpreting the results from these single studies as they are often underpowered due to inadequate sample sizes. The present meta-analysis, which incorporated 6 independent case-control studies, was designed to evaluate whether or not rs243865 MMP-2 polymorphism modifies the risk of developing AMD. In comparison to past single studies, our analysis included a large sample size, 1,682 AMD patients and 2,295 healthy subjects, with attendant increased statistical power. Also, a quality evaluation system was included to minimise the potential for bias. Based on this approach, our most significant finding was that the 16q13-q21 rs243865 MMP-2 polymorphism is not a risk factor for developing AMD.

MMPs and tissue regulators have a central role in the deposition of extracellular matrix accumulations in several diseases [31–34]. It has been reported that MMP-2, MMP-3, and MMP-9 could be related to the pathogenesis of AMD. MMP-2, also known as gelatinase A, participates in the degradation of extracellular matrix components like collagen and laminin [34–36]. The rs243865 MMP-2 variant is located in the promoter region of the gene, where it causes a modification in MMP-2 transcription due to the variant T allele alteration in the Sp1-type promoter site [13]. The C to T allelic variation disrupts the Sp1 binding site in the promoter region and leads to significantly lower transcriptional activity. Therefore, the T allele has a markedly lower activity (50%) than the C allele. Several authors have reported that the dysregulation of MMP-2 synthesis leads to the formation of deposits in the basement membrane of the retinal pigment epithelium that, in turn, could contribute to drusen formation, and therefore to AMD development [34–36]. Moreover, an increased amount of inactive MMP-2 has been found in the subretinal space of AMD patients compared to that in normal human donor eyes [37]. This diminished level of active MMP-2 could be related to a reduction in the remodelling and accumulation of basal laminar deposits in AMD subjects [38].

Although the MMP pathway could play an important role in AMD development, the results of our study suggest that the rs243865 MMP-2 polymorphism was not associated with an increased risk of developing AMD. In addition, no statistically significant differences were attributable to either ethnicity or age. Therefore, other genetic and/or environmental factors might be more crucial than the rs243865 MMP-2 polymorphism in the susceptibility of AMD development. In this study, we analysed only one selected polymorphism within the MMP-2 gene; therefore, our results do not exclude the involvement of other MMP-2 gene variations in AMD pathogenesis.

It is necessary to emphasize the limitations in our meta-analysis. First, only English-language articles were analysed, which may have limited the number of studies included in our meta-analysis. Second, not all the studies reported adjusted ORs, and adjustments for the level of confounders such as geographic distribution, type of AMD, ethnicity, age, and gender varied among them. Third, we did not specify our results according to AMD type due to the small sample size that could have reduced the statistical power. Finally, our meta-analysis was focused mainly on the Caucasian population as the Asian population provided only a relatively small sample size. Nevertheless, we have been able to overcome the statistical limitations of small sample size and homogeneous sample composition that is commonly encountered in allelic genetic association studies. Even with these limitations, this meta-analysis contributes significantly to our understanding of AMD because it is the first to clarify the risk of development associated with the rs243865 MMP-2 polymorphism. Multiple GWAS and meta-analysis studies for AMD have been published, but to our knowledge, none of them included in an analysis of the rs243865 MMP-2 polymorphism.

## Conclusions

In conclusion, our meta-analysis provides reliable evidence that rs243865 MMP-2 polymorphism is unlikely to be a risk factor for AMD development. Further investigations analysing the combined effect of genes and the environment may improve our current understanding of the association between the rs243865 or other MMP-2 polymorphisms and the risk of developing AMD, as well as the clinical and biological implications of other risk factors.

## Supporting information

S1 Table Checklist for meta-analysis of genetic association studies (PLOS ONE approach). (DOC)

S2 Table Search terms used for systematic literature search. (DOCX)

S3 Table Study quality assessment by the Newcastle-Ottawa Quality Assessment Scale. (DOCX)

## References

[1] Wang JJ, Foran S, Mitchell P. Age-specific prevalence and causes of bilateral and unilateral visual impairment in older Australians: the Blue Mountains Eye Study. Clin Experiment Ophthalmol. 2000 8;28(4):268–73. 11021555 10.1046/j.1442-9071.2000.00315.x

[2] Wong WL, Su X, Li X, Cheung CMG, Klein R, Cheng C-Y, et al Global prevalence of age-related macular degeneration and disease burden projection for 2020 and 2040: a systematic review and meta-analysis. Lancet Glob Health. 2014 2;2(2):e106–116. 10.1016/S2214-109X(13)70145-1 25104651

[3] Jonasson F, Arnarsson A, Eiríksdottir G, Harris TB, Launer LJ, Meuer SM, et al Prevalence of age-related macular degeneration in old persons: Age, Gene/environment Susceptibility Reykjavik Study. Ophthalmology. 2011 5;118(5):825–30. 10.1016/j.ophtha.2010.08.044 21126770 PMC3087833

[4] Ferris FL, Wilkinson CP, Bird A, Chakravarthy U, Chew E, Csaky K, et al Clinical classification of age-related macular degeneration. Ophthalmology. 2013 4;120(4):844–51. 10.1016/j.ophtha.2012.10.036 23332590 PMC11551519

[5] Zarbin MA. Current concepts in the pathogenesis of age-related macular degeneration. Arch Ophthalmol. 2004 4;122(4):598–614. 10.1001/archopht.122.4.598 15078679

[6] Ratnapriya R, Chew EY. Age-related macular degeneration-clinical review and genetics update. Clin Genet. 2013 8;84(2):160–6. 10.1111/cge.12206 23713713 PMC3732788

[7] Seitzman RL, Mahajan VB, Mangione C, Cauley JA, Ensrud K, Stone KL, et al Estrogen receptor alpha and matrix metalloproteinase 2 polymorphisms and age-related maculopathy in older women. American Journal of Epidemiology. 2008 5 15;167(10):1217–25. 10.1093/aje/kwn024 18359774

[8] Ortak H, Demir S, Ates O, Benli I, Sogut E, Sahin M. The Role of MMP2 (-1306C > T) and TIMP2 (-418 G > C) Promoter Variants in Age-related Macular Degeneration. Ophthalmic Genetics. 2013 12;34(4):217–22. 10.3109/13816810.2013.781192 23536957

[9] Liutkeviciene R, Lesauskaite V, Zaliaduonyte-Peksiene D, Sinkunaite-Marsalkiene G, Zaliuniene D, Mizariene V, et al Role of MMP-2 (-1306 C/T) Polymorphism in Age-Related Macular Degeneration. Ophthalmic Genet. 2016;37(2):170–6. 10.3109/13816810.2015.1020556 26333112

[10] Cheng J, Hao X, Zhang Z. Risk of macular degeneration affected by polymorphisms in Matrix metalloproteinase-2: A case-control study in Chinese Han population. Medicine (Baltimore). 2017 11;96(47):e8190. 29381911 10.1097/MD.0000000000008190 PMC5708910

[11] Liutkeviciene R, Lesauskaite V, Sinkunaite-Marsalkiene G, Simonyte S, Zemaitiene R, Kriauciuniene L, et al MMP-2 Rs24386 (C—>T) gene polymorphism and the phenotype of age-related macular degeneration. Int J Ophthalmol. 2017;10(9):1349–53. 10.18240/ijo.2017.09.03 28944191 PMC5596217

[12] Liutkeviciene R, Vilkeviciute A, Borisovaite D, Miniauskiene G. Association of exudative age-related macular degeneration with matrix metalloproteinases-2 (-1306 C/T) rs243865 gene polymorphism. Indian Journal of Ophthalmology. 2018 4;66(4):551–7. 10.4103/ijo.IJO_1050_17 29582818 PMC5892060

[13] Price SJ, Greaves DR, Watkins H. Identification of novel, functional genetic variants in the human matrix metalloproteinase-2 gene: role of Sp1 in allele-specific transcriptional regulation. J Biol Chem. 2001 3 9;276(10):7549–58. 10.1074/jbc.M010242200 11114309

[14] Vu TH, Werb Z. Matrix metalloproteinases: effectors of development and normal physiology. Genes Dev. 2000 9 1;14(17):2123–33. 10970876 10.1101/gad.815400

[15] Page-McCaw A, Ewald AJ, Werb Z. Matrix metalloproteinases and the regulation of tissue remodelling. Nat Rev Mol Cell Biol. 2007 3;8(3):221–33. 10.1038/nrm2125 17318226 PMC2760082

[16] Little J, Higgins JPT, Ioannidis JPA, Moher D, Gagnon F, von Elm E, et al STrengthening the REporting of Genetic Association Studies (STREGA): an extension of the STROBE statement. PLoS Med. 2009 2 3;6(2):e22 10.1371/journal.pmed.1000022 19192942 PMC2634792

[17] Age-related macular degeneration | Guidance and guidelines | NICE [Internet]. [cited 2018 Nov 2]. https://www.nice.org.uk/guidance/ng82/chapter/recommendations

[18] Bird AC, Bressler NM, Bressler SB, Chisholm IH, Coscas G, Davis MD, et al An international classification and grading system for age-related maculopathy and age-related macular degeneration. The International ARM Epidemiological Study Group. Surv Ophthalmol. 1995 4;39(5):367–74. 7604360 10.1016/s0039-6257(05)80092-x

[19] Stang A. Critical evaluation of the Newcastle-Ottawa scale for the assessment of the quality of nonrandomized studies in meta-analyses. Eur J Epidemiol. 2010 9;25(9):603–5. 10.1007/s10654-010-9491-z 20652370

[20] Cochran WG. The Combination of Estimates from Different Experiments. Biometrics. 1954;10(1):101–29.

[21] Higgins JPT, Thompson SG, Deeks JJ, Altman DG. Measuring inconsistency in meta-analyses. BMJ. 2003 9 6;327(7414):557–60. 10.1136/bmj.327.7414.557 12958120 PMC192859

[22] Review Manager (RevMan) [Computer program]. Version 5.3. Copenhagen: The Nordic Cochrane Centre, The Cochrane Collaboration, 2014.

[23] Cochrane Handbook for Systematic Reviews of Interventions [Internet]. [cited 2018 Nov 2]. http://handbook-5-1.cochrane.org

[24] Fritsche LG, Chen W, Schu M, Yaspan BL, Yu Y, Thorleifsson G, et al Seven new loci associated with age-related macular degeneration. Nat Genet. 2013 4;45(4):433–9, 439–2. 10.1038/ng.2578 23455636 PMC3739472

[25] Fritsche LG, Igl W, Bailey JNC, Grassmann F, Sengupta S, Bragg-Gresham JL, et al A large genome-wide association study of age-related macular degeneration highlights contributions of rare and common variants. Nat Genet. 2016 2;48(2):134–43. 10.1038/ng.3448 26691988 PMC4745342

[26] Black JRM, Clark SJ. Age-related macular degeneration: genome-wide association studies to translation. Genet Med. 2016 4;18(4):283–9. 10.1038/gim.2015.70 26020418 PMC4823638

[27] Francis PJ, Schultz DW, Hamon S, Ott J, Weleber RG, Klein ML. Haplotypes in the Complement Factor H (CFH) Gene: Associations with Drusen and Advanced Age-Related Macular Degeneration. PLOS ONE. 2007 11 28;2(11):e1197 10.1371/journal.pone.0001197 18043728 PMC2077927

[28] Grassmann F, Heid IM, Weber BHF, International AMD Genomics Consortium (IAMDGC). Recombinant Haplotypes Narrow the ARMS2/HTRA1 Association Signal for Age-Related Macular Degeneration. Genetics. 2017;205(2):919–24. 10.1534/genetics.116.195966 27879347 PMC5289859

[29] Johnson LV, Ozaki S, Staples MK, Erickson PA, Anderson DH. A potential role for immune complex pathogenesis in drusen formation. Exp Eye Res. 2000 4;70(4):441–9. 10.1006/exer.1999.0798 10865992

[30] Hageman GS, Mullins RF. Molecular composition of drusen as related to substructural phenotype. Mol Vis. 1999 11 3;5:28 10562652

[31] Belkhiri A, Richards C, Whaley M, McQueen SA, Orr FW. Increased expression of activated matrix metalloproteinase-2 by human endothelial cells after sublethal H2O2 exposure. Lab Invest. 1997 11;77(5):533–9. 9389796

[32] Dollery CM, Libby P. Atherosclerosis and proteinase activation. Cardiovasc Res. 2006 2 15;69(3):625–35. 10.1016/j.cardiores.2005.11.003 16376322

[33] Peten EP, Garcia-Perez A, Terada Y, Woodrow D, Martin BM, Striker GE, et al Age-related changes in alpha 1- and alpha 2-chain type IV collagen mRNAs in adult mouse glomeruli: competitive PCR. Am J Physiol. 1992 11;263(5 Pt 2):F951–957. 10.1152/ajprenal.1992.263.5.F951 1443183

[34] Atkinson SJ, Patterson ML, Butler MJ, Murphy G. Membrane type 1 matrix metalloproteinase and gelatinase A synergistically degrade type 1 collagen in a cell model. FEBS Lett. 2001 3 2;491(3):222–6. 11240131 10.1016/s0014-5793(01)02204-9

[35] Elliot SJ, Catanuto P, Espinosa-Heidmann DG, Fernandez P, Hernandez E, Saloupis P, et al Estrogen receptor beta protects against in vivo injury in RPE cells. Exp Eye Res. 2010 1;90(1):10–6. 10.1016/j.exer.2009.09.001 19799898 PMC2789169

[36] Liutkeviciene R, Lesauskaite V, Sinkunaite-Marsalkiene G, Zaliuniene D, Zaliaduonyte-Peksiene D, Mizariene V, et al The Role of Matrix Metalloproteinases Polymorphisms in Age-Related Macular Degeneration. Ophthalmic Genet. 2015 6;36(2):149–55. 10.3109/13816810.2013.838274 24079541

[37] Plantner JJ, Jiang C, Smine A. Increase in interphotoreceptor matrix gelatinase A (MMP-2) associated with age-related macular degeneration. Exp Eye Res. 1998 12;67(6):637–45. 10.1006/exer.1998.0552 9990329

[38] Guo L, Hussain AA, Limb GA, Marshall J. Age-dependent variation in metalloproteinase activity of isolated human Bruch’s membrane and choroid. Invest Ophthalmol Vis Sci. 1999 10;40(11):2676–82. 10509665

