# Supplementary material for: Meta-analysis of the rs243865 MMP-2 polymorphism and age-related macular degeneration risk
Source: PLoS One. 2019 Mar 7;14(3):e0213624. doi: 10.1371/journal.pone.0213624 (PMC6405106; doi:10.1371/journal.pone.0213624)
Supplement: S2 Table — (DOCX) [file pone.0213624.s002.docx]

S2 Table. Search terms used for systematic literature search

"matrix metalloproteinase-2" AND (“polymorphism” OR “rs243865” OR ” -1306C>T”) AND ("age-related macular degeneration" OR “macular degeneration” OR “AMD” OR “maculopathy”
