# Supplementary material for: Meta-analysis of the rs243865 MMP-2 polymorphism and age-related macular degeneration risk
Source: PLoS One. 2019 Mar 7;14(3):e0213624. doi: 10.1371/journal.pone.0213624 (PMC6405106; doi:10.1371/journal.pone.0213624)
Supplement: S3 Table — (DOCX) [file pone.0213624.s003.docx]

S3 Table. Study quality assessment by the Newcastle-Ottawa Quality Assessment Scale

| **Authors, year** | NEWCASTLE OTTAWA QUALITY ASSESSMENT SCALE  Case Control Studies | | | | | |
| --- | --- | --- | --- | --- | --- | --- |
|  | **Country** | **AMD patients** | **Healthy subjects** | **Selection** | **Comparability** | **Exposure** |
| Seitzman et al, 2008 | USA | 434 | 456 | ★★★★ | ★★ | ★ |
| Ortak et al, 2013 | Turkey | 144 | 172 | ★★★★ | ★★ | ★ |
| Liutkeviciene et al, 2016 | Lithuania | 387 | 682 | ★★★ | ★★ | ★ |
| Cheng et al, 2017 | China | 126 | 141 | ★★★★ | ★ | ★ |
| Liutkeviciene et al, 2017 | Lithuania | 324 | 526 | ★★★ | ★★ | ★ |
| Liutkeviciene et al, 2018 | Lithuania | 267 | 318 | ★★★ | ★★ | ★ |
